# Embodied Music Cognition: Trouble Ahead, Trouble Behind

**DOI:** 10.3389/fpsyg.2016.01891

**Published:** 2016-12-12

**Authors:** Jakub R. Matyja

**Affiliations:** Polish Academy of Sciences, Institute of Philosophy and SociologyWarsaw, Poland

**Keywords:** embodied music cognition, embodied cognition, music cognition, radical embodied cognitive science, music perception, psychology of music

These are interesting times for the Embodied Music Cognition (EMC) framework. Briefly summarized, EMC relies upon the hypothesis that embodied sensorimotor engagement is essential to both production and perception of music (Leman, 2008; Leman and Maes, 2014). This hypothesis, while suitable for accommodating different research methods used in empirical musicology (Godøy and Leman, 2010), may be regarded as inherently rather abstract. In the light of recent critical accounts on the foundations of embodied approaches to cognition as such (e.g., Goldinger et al., 2016, point out that premises like “perception is influenced by the body” are unacceptably vague), EMC hypothesis begs for further specification. Anticipating it, Leman and Maes (2014) suggested that the process of disambiguating what EMC hypothesis stands for, may take two forms. Firstly, the key would be to show that “embodiment plays a core role in an interconnected network of cognitive and emotive functions” and this network would be “further crucial in affect processing, conceptualization, tool use, and the entire array of functions needed to make sense of music” (Leman and Maes, 2014, p. 237). Second option, which as Leman and Maes (2014, p. 237) suggest “narrows down the perspective in order to find empirical evidence for a specific hypothesis,” would be to show that “the effect of action is essential to making sense of music.” In order to constructively contribute to the development of Leman and Maes' intuitions as to where EMC research should go, in what follows I will consider some challenges that may be of interests to both theoretically and empirically minded researchers.

## From metaphors to viable models

Leman and Maes' (2014, p. 237) challenge of establishing the “core role” that a body plays in the “network of cognitive and emotive functions” is as interesting as it is speculative. Consider Maes (2016) proposal for grounding EMC in terms of dynamic systems. Maes collects empirical evidence and radicalizes the (already vague) EMC basic principle by arguing that “music perception is a dynamic process firmly rooted in the natural disposition of sounds and the human auditory and motor system” (Maes, 2016, p. 1). The basic principle of EMC is “radicalized” by showing the ways in which it is *consistent* with recent research on predictive coding and dynamic systems in cognitive science. However, as researchers (e.g., Mole and Klein, 2010) convincingly argue, consistency itself is not enough to make a case in support of a given hypothesis. The key idea behind the so-called *consistency fallacy*: a given body of data cannot be stated as consistent with a particular theory (e.g., of EMC) just for the sake of *consistency* with that theory. For a body of data to provide evidence for the theory something else is needed: the data must not only be consistent with the hypothesis, they must also count against the contradiction of that hypothesis.

It is unclear, however, how a particular set of empirical data [e.g., neuroscientific data suggesting that listening to music triggers the motor responses in the brain (Bangert and Altenmüller, 2003 quoted in Maes et al., 2014)] may count against the standard “brain-centered” approaches to music cognition (for discussion, see Matyja and Schiavio, 2013). The positive proposal here would be to focus upon establishing the basic requirements for what distinguishes the “embodied” aspects of musical processing from the “disembodied” ones. A possible strategy here would be to move beyond the phenomenological metaphors of how “body shapes musical processing,” and put some more research effort into accommodating the physiological (e.g., bloodflow, skin conductivity as well as biochemical and psychoneuroimmunological responses to music, see Fancourt et al., 2014; Hodges, 2016) aspects of how music perception influences the *actual* workings of a human body.

## Narrowing down the EMC perspective

The research paradigm of mechanistic explanation (e.g., Craver, 2007) may provide some useful tools for the viable reconstruction of present works on embodied (music) cognition (e.g., Matyja, 2015; Matyja and Dolega, 2015). Now, it is safe to assume that musical perception results from operations of different hierarchically organized levels of sub-mechanisms that constitute a living organism. The key idea behind mechanistic explanation lies in the identification of the parts of a mechanism (e.g., human body) and establishing in how they interact with each other in such a way that produces a given phenomenon in question (e.g., music perception). Only after discovering those mechanisms and their internal organization and interactions between them, one can theorize about the role functions (e.g., Craver, 2001) that they serve for a living organism interacting with music. While I understand that it is easier said than done, my key contention is that the future (and explanatory force) of EMC lies in moving beyond the metaphors and going in line with methodological naturalism. That would require, however, showing *how* (step-by-step) the phenomena that are hypothesized to be embodied are actually constituted the workings of a body. Note that this strategy is different from currently proposed approaches. The issues of how empirical *data* relates to *theory* of EMC has been recently of interests for some researchers (e.g. Schiavio, 2014; Maes, 2016). For instance, Maes (2016, p. 2)—inspired by Mahon and Caramazza (2008)—briefly underlines the worry regarding the drawing of (theoretical) conclusions from available empirical data in EMC research. However, after mentioning the worry he goes on suggesting the need for “collecting further empirical evidence and computational models to substantiate the role of interaction in the embodied thesis in the domain of music perception and performance” (Maes, 2016, p. 2). The problem is, however, that the reference to present or future data itself is not enough to “substantiate” the initial EMC hypothesis.

Recall the distinction between *data* and *phenomena* in philosophy of science (Bogen and Woodward, 1988). Roughly speaking, theories (e.g., EMC hypothesis) explain particular phenomena. A phenomenon (e.g., body affecting musical processing) is a repeatable type of event or product (e.g., it occurs when human perceive music) that is not readily observable. Conversely, empirical data (e.g., Maes et al., 2014 for an overview) come from individual research instances that have complex causes (that are idiosyncratic to particular experimental situations). Now, the crux of the matter is that data may serve as evidence for the phenomenon, but the mere reference to the acquired experimental data is not enough to substantiate the EMC framework. Given the initial vagueness of the guiding hypotheses of EMC, it is not exactly clear how any amount of future empirical data can help to refine it. Conversely, EMC research frameworks needs a clear hypothesis—a hypothesized multileveled model of how the body actually influences musical processing (and does so in a way that cannot be accounted by the preceding “disembodied” perspectives) to begin with.

## Author contributions

The author confirms being the sole contributor of this work and approved it for publication.

## Funding

JM's involvement in this project was funded by National Science Centre (Poland) research grant under the decision DEC-2014/14/E/HS1/00803.

### Conflict of interest statement

The author declares that the research was conducted in the absence of any commercial or financial relationships that could be construed as a potential conflict of interest.
